# Metagenomic source tracking after microbiota transplant therapy

**DOI:** 10.1080/19490976.2025.2487840

**Published:** 2025-04-14

**Authors:** Susan L. Hoops, Daphne Moutsoglou, Byron P. Vaughn, Alexander Khoruts, Dan Knights

**Affiliations:** a Department of Computer Science and Engineering, University of Minnesota, Minneapolis, MN, USA; b Biotechnology Institute, University of Minnesota, Minneapolis, MN, USA; c Gastroenterology Section, Minneapolis VA Health Care System, Minneapolis, MN, USA; d Department of Medicine, University of Minnesota, Minneapolis, MN, USA; e Division of Gastroenterology, University of Minnesota, Minneapolis, MN, USA; f Center for Immunology, University of Minnesota, Minneapolis, MN, USA

**Keywords:** Metagenomics, ulcerative colitis, microbiota engraftment, shotgun sequencing, whole-genome sequencing, microbiota transplant therapy, fecal microbiota transplant, metagenome-assembled genome

## Abstract

Reliable engraftment assessment of donor microbial communities and individual strains is an essential component of characterizing the pharmacokinetics of microbiota transplant therapies (MTTs). Recent methods for measuring donor engraftment use whole-genome sequencing and reference databases or metagenome-assembled genomes (MAGs) to track individual bacterial strains but lack the ability to disambiguate DNA that matches both donor and patient microbiota. Here, we describe a new, cost-efficient analytic pipeline, MAGEnTa, which compares post-MTT samples to a database comprised MAGs derived directly from donor and pre-treatment metagenomic data, without relying on an external database. The pipeline uses Bayesian statistics to determine the likely sources of ambiguous reads that align with both the donor and pre-treatment samples. MAGEnTa recovers engrafted strains with minimal type II error in a simulated dataset and is robust to shallow sequencing depths in a downsampled dataset. Applying MAGEnTa to a dataset from a recent MTT clinical trial for ulcerative colitis, we found the results to be consistent with 16S rRNA gene SourceTracker analysis but with added MAG-level specificity. MAGEnTa is a powerful tool to study community and strain engraftment dynamics in the development of MTT-based treatments that can be integrated into frameworks for functional and taxonomic analysis.

## Introduction

Fecal microbiota transplantation (FMT) emerged in response to the challenge posed by increasingly difficult recurrent *Clostridioides difficile* infections. Over the past decade the crude FMT preparations made by individual clinical practitioners have largely transitioned to standardized FMT-based products manufactured in centralized facilities, and recently two FMT-based products received the approval of the US Food and Drug Administration.^1,2^ The success of FMT in breaking the recurrent *C. difficile* infection cycle has also stimulated interest in using it to target the gut microbiome for a multitude of other indications, ranging from inflammatory bowel disease to optimization of cancer checkpoint immunotherapy to autism. However, unlike recurrent *C. difficile* infections, where a single administration of a single inoculum of healthy donor FMT is generally sufficient to repair a microbiome decimated by multiple cycles of antibiotics, the goal of restructuring a stable but presumably unhealthy microbiota in most indications other than recurrent *C. difficile* infections is generally much more challenging. The dosing regimens for such indications are typically much more elaborate and may involve antibiotic preconditioning and/or repeated administrations of donor microbiota.^3,4^ This complexity is acknowledged by the term *microbiota transplant therapy* or MTT, which sets it apart from a simple FMT.^4^

The US Food and Drug Administration classifies donor microbiota as live biotherapeutic products. However, the pharmacology of live biotherapeutic products is clearly distinct from that of small molecules and protein biologics.^5^ One of the critical aspects of the pharmacology of any drug is its pharmacokinetics, which describes what happens to the drug in the body. In the case of live biotherapeutic products, pharmacokinetics requires an assessment of donor microbiota engraftment and retention over time. Donor microbiota engraftment can be estimated quantitatively as a proportion or percentage of donor-derived microbes in the post-treatment microbial composition and qualitatively as the types and variety of microbiota successfully retained by the recipient following treatment. SourceTracker is one tool for evaluating microbial engraftment.^6^ It provides a Bayesian estimation of pre- and post-FMT donor similarity by comparing donor and pre-FMT samples (sources) and post-FMT samples (sink). We have applied SourceTracker to 16S rRNA gene data to describe the donor microbiota engraftment kinetics following administration of standardized FMT via oral capsules or colonoscopy in treatment of recurrent *C. difficile* infections, a condition with stark differences between the donor and recipient microbiota.^7,8^ More recently, we have shown SourceTracker to be successful in distinguishing MTT and placebo intervention in a randomized trial in participants with ulcerative colitis.^9^ However, 16S rRNA gene sequencing does not allow for strain-level specificity. Previous studies have also modeled engraftment using both 16S rRNA gene and whole-genome sequencing (WGS) data by community alpha and beta diversity or by measuring overlapping taxonomies appearing in the recipient following microbiota administration. These approaches provide only a limited model of underlying strain engraftment.

WGS provides the potential for strain-level engraftment measurements. Some longitudinal studies and robust meta-analyses of FMT data have assessed engraftment from WGS data by establishing metrics for strain sharing between donors and recipients using metagenome assembled genomes (MAGs) binned into MAG-derived strains.^10–12^ Alternatively, single nucleotide polymorphisms and single nucleotide variant profiles have been used to detect and compare strains shared between samples.^3,13^ These methods do perform source tracking at the strain and MAG level, and in some cases they do provide estimates of overall mixing proportions, but they do not provide Bayesian redistribution of ambiguous reads when mapping reads to MAGs. Another method, Meta-SourceTracker,^14^ applies Bayesian mixture modeling to deconvolve source assignments for shared species across sources, treating taxonomic features as the mixing components in the same manner as the original SourceTracker model. This approach has the benefit of estimating total mixing proportions from potentially unknown environments and could be adapted to provide strain-level or MAG-level read-by-read source assignments, although it does not allow Bayesian redistribution of taxonomically ambiguous sequences to the most likely source environment. Furthermore, the statistical model underlying mixture modeling methods such as Meta-SourceTracker and SourceTracker assume that the original source mixtures of taxa or MAGs have not changed over time since the environmental mixing occurred. This assumption is less applicable to MTT-related data, where we expect that some donor strains will engraft at a higher rate than others, changing the original mixing ratios of strains in the donor microbiota.^10–12^

In the present work, we describe an analytic pipeline, *MAGEnTa*, which expands on the existing MAG-based approaches by using unbinned, de-novo MAGs as the reference database for the donor and patient to quantitatively evaluate overall donor community engraftment at the strain level. Furthermore, this method resolves the assignment of ambiguous DNA sequences in the estimation of engraftment by redistribution of those read counts to the most likely sources using Bayesian statistics. This de-novo MAG approach, like prior MAG-based approaches, avoids discarding reads that do not map to preexisting reference database.^14^ Binning approaches that rely on assembling near-complete MAGs may be prone to similar data loss due to uneven complexity of human microbial communities, leading to many unassembled reads from rare and therefore incomplete strains.^15^ Our MAG-based approach aims to minimize information loss in quantifying overall microbial community engraftment, while also enabling further qualitative exploration of pharmacokinetics of engrafted strains.

## Results

### MAGEnTa pipeline for accurate measurement of microbial engraftment

**Figure 1.**
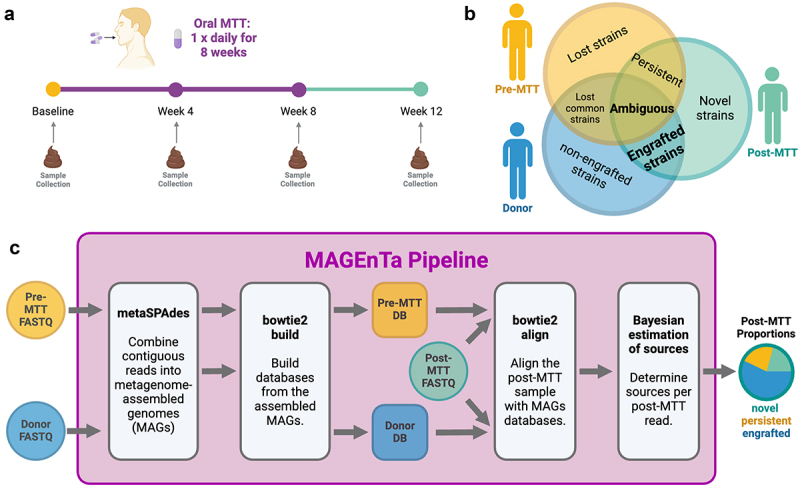
(a) Schema for randomized, double-blinded, placebo-controlled trial of microbiota transplant therapy (MTT) for participants with mild-to-moderate ulcerative colitis. Participants were administered placebo capsules or encapsulated MTT from a healthy donor daily for 8 weeks. Stool samples for metagenomic sequencing were collected from participants at baseline (week 0), and weeks 4, 8, and 12. (b) A visual depiction of pre-treatment (yellow), donor (blue), and post-treatment (green) strains as a Venn Diagram. While the engrafted donor strains constitute the focus of our manuscript, further information on MTT impacts can also be found in determining the likelihood of ambiguous strains being engrafted or persistent. (c) A visual depiction of the MAGEnTa pipeline. We begin with using metaSpades and Bowtie 2 to construct metagenome assembled genomes (MAGs) databases from the pre-mtt and donor samples. We then align the post-MTT sample with these databases to determine which reads match the donor and the pre-mtt (baseline) samples. Ambiguous reads matching both databases are evaluated by a Bayesian estimation to assign the likely source. Read source outputs are combined into proportions, assigning each read to engrafted, persistent, or novel/unknown (unmapped). This figure was created in BioRender.com. DB (database), MAGs (metagenome assembled genomes), MTT (microbiota transplant therapy).

The immediate objective that led us to the development of this analytic pipeline was to distinguish donor and recipient bacterial strains, which facilitate accurate measurements of donor microbiota engraftment in an MTT trial for ulcerative colitis. In contrast to the decimated microbiomes of patients with recurrent *C. difficile* infections due to repeated antibiotic administrations, the microbiome changes associated with ulcerative colitis are relatively mild, which potentially may complicate assessment of MTT-induced changes. The schematic for the ulcerative colitis trial involving 27 participants is shown in Figure 1(a). In principle, the MTT protocol in this case results in a collision of two microbiomes within the participant’s intestine, which is also open to acquiring bacterial novel strains from the environment. Figure 1(b) shows the Venn diagram that illustrates the potential attribution of strains to their original sources when we analyze samples in the recipient. The schematic for our MAGEnTa analytic pipeline is shown in Figure 1(c).

MAGEnTa recovers engrafted microbes with strain-level specificity using nucleotide sequence alignment with MAGs for fast and reliable estimation of microbial sources. Following quality control and trimming for sequencing artifacts, metagenomic sequencing reads from the donor and pre-treatment samples are evaluated by metaSPAdes to obtain MAGs for each donor and pre-treatment sample.^16^ These MAGs are converted to Bowtie 2 databases for each donor and pre-treatment sample, using the genomic sequence scaffolds as the basis for each database. We then evaluate the source of each read in the post-treatment samples by aligning the reads with their respective donor and pre-treatment databases. Ambiguous reads, or those aligning with both the donor and pre-treatment databases, are allocated via a Bayesian estimator as either a donor (engrafted strains) or pre-treatment (persistent strains) database match. The alignment results provide a quantitative engraftment value based on the total reads aligning with the donor database, as well as an estimate of persistent reads and novel strains. These donor MAGs can subsequently be binned or aligned with a reference database to qualitatively evaluate the taxonomy of the strains which engrafted following MTT.

The MAGEnTa pipeline assumes reads are pre-filtered for quality control and adapter trimming. We used KneadData^17^ to conduct quality control on the raw sequencing files, however any comparable tool would work so long as FASTA or FASTQ formatted files are produced. We recommend trimming adapter sequences and filtering for low quality, although a relatively low threshold is often sufficient for short shotgun reads.^18^ These filtered FASTQ read files serve as the input for our pipeline.

Filtered reads are passed into the start of the MAGEnTa pipeline, combining contiguous reads into longer contigs via the metaSPAdes metagenomic assembler. Building a deBruijn graph from reads using SPAdes,^19^ the graph is simplified, removing bulges and long tips, into an “assembly graph” that when traversed, results in long, individual gene fragments.^20^ These traversed fragments form scaffolds representing strain-level DNA fragments from the microbes contained in the sample. To ensure the uniqueness and efficacy of these scaffolds, low coverage edges in the graph are disconnected to preserve as much information about rare strains as possible without biasing the consensus contig and resulting scaffold. Resulting scaffolds from metaSPAdes will constitute the databases used in tracking engraftment.

Bowtie 2 constructs index files from sequences using the bowtie2-build command. An index constructed by Bowtie 2 can effectively be used in place of a reference database for alignment. Having constructed MAGs from the donor and pre-treatment samples, we index these scaffolds to form unique databases in Bowtie 2 format. We can then trace sources of reads in the post-treatment samples by aligning reads with the unique databases representing the donor and the pre-treatment samples independently.

Using Bowtie 2 alignment we obtain alignment matches with the donor and pre-treatment samples, respectively. Comparing these two alignment files with one another and the original reads, we bin all post-treatment reads into bins of uniquely donor-aligned, uniquely pre-treatment-aligned, ambiguously aligned with both databases, and unmapped. We assume unmapped reads containing sequencing errors come from low-abundance strains that were not assembled into MAGs, or represent novel strains introduced to the patient by their diet and environment. To adjust for the first two types of unmapped reads, we first align the donor and pre-treatment samples with their own MAGs database. This provides an expected fraction of unmapped reads due to sequencing error and missing MAG content. Remaining unmapped reads can therefore be characterized as novel strains. Reads aligning with both the donor and pre-treatment databases are categorized as ambiguous reads. We then apply a Bayesian approach to the fraction of ambiguous reads to distribute them to the most likely sources.

To allocate ambiguous reads to their sources, we apply Bayes’ theorem. Given two possible source databases, donor microbiota and the original pre-treatment patient microbiota, let us use ***p***_*i = 1 … n*_ ∈{true, false} and ***d***_*i = 1 … n*_ ∈{true, false} as Boolean variables to represent the observations of which reads aligned to patient database and donor database, respectively. Then, ***p***_*i*_ indicates that read *i* aligned to the patient database, and ***d***_*i*_ that read *i* aligned to the donor database. We use separate Boolean variables ***p*** and ***d*** for observed alignment to the two different databases because they can both be true for a given read. Let ***z***_*i = 1 … n*_ ∈{true, false} represent the hidden Boolean variables of true donor source assignment events for the reads, where ***z***_*i*_ is true when the true source is the donor, and false when the true source is the patient. The posterior probability that the true source assignment of an ambiguous read *i* is the donor is given in Equation 1:

$$P(z_{i}|p_{i},d_{i})=\frac{P(p_{i},d_{i}|z_{i})P\left(z_{i}\right)}{P\left(p_{i},d_{i}\right)}$$

The denominator can be expanded as follows, in Equation 2:

$$P(z_{i}|p_{i},d_{i})=\frac{P(p_{i},d_{i}|z_{i})P\left(z_{i}\right)}{P\left(p_{i},d_{i}|z_{i}\right)P\left(z_{i}\right)+P(p_{i},d_{i}|\neg z_{i})P\left(\neg z_{i}\right)}$$

First, we estimate the prior probabilities $P\left(z_{i}\right)$ and $P\left(\neg z_{i}\right)$ from the proportions of sample reads uniquely assigned to donor and patient, respectively. We then calculate the likelihood $P(p_{i},d_{i}|z_{i})$ that a read from the donor database aligns to both the patient database and donor database by aligning reads from the original donor sample both against its own MAG database and against the patient MAG database, and calculating the proportion of reads that aligned with both. A similar calculation is performed to obtain $P(p_{i},d_{i}|\neg z_{i})$ by aligning the original pre-treatment patient sample reads with both databases. From these equations, we estimate the proportion of engrafted and persistent reads among the ambiguously aligned reads.

Finally, the pipeline produces quantitative values for engrafted, persistent, and novel proportions or percentages in the recipient sample. Engrafted reads are listed in a separate file with their matching MAGs, enabling further qualitative analysis via binning, taxonomic classification, or diversity analysis.

### Validation of the MAGEnTa pipeline by simulated trials

**Figure 2.**
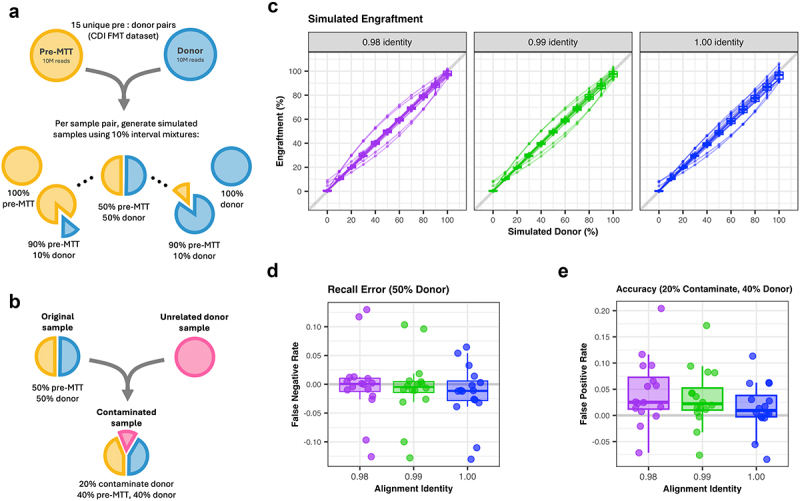
(a) Creation of 15 simulated datasets totaling 165 samples. For each of 15 donor and pre-mtt pairs in a CDI study, we obtain 11 samples comprised of different percentages of the donor and pre-microbiota transplant therapy (MTT) material. All samples are an even depth of 10 million reads. (b) From the 50% donor and 50% pre-mtt simulated samples, we also simulated the inclusion of an external, “contaminate” source. For this simulation, our external source is another healthy donor unrelated to the original donor and pre-MTT pair. This simulation seeks to represent other microbes introduced to the patient by external means such as food or environment. (c) Engraftment resulting from 15 simulations, displaying simulated (x-axis) vs. observed (y-axis), separated and colored by alignment identity. The expected outcome would be an x=y line as shown in gray, which the observed results closely follow. (d) The recall error of the algorithm per identity with the 50% donor and 50% pre samples, showing the false negative rate (type II error) per simulation, grouped by identity. Less stringent identity has a slightly lower type II error rate. (e) The accuracy error when 20% of the simulated 50% donor sample comes from a contaminate source in the form of an unrelated donor sample. The more stringent the identity, the fewer false positives (type I error).

To validate our algorithm, we simulated engraftment at 10% intervals from 0% donor to 100% donor using paired donor and pre-treatment samples in a recurrent *C. difficile* infection study (Figure 2(a)).^13^ Applying the algorithm to each of our simulated samples we found a near perfect recovery of engraftment at varying alignment identity (Figure 2(c)). To evaluate the sensitivity of the algorithm to alignment match parameters, we compared results when using 98% identity read alignment, 99% identity read alignment, and 100% identity read alignment. We could expect 98% to introduce some amount of error as there may be closely related reads which falsely align with other MAGs. Conversely, we expected 100% identity results to be more susceptible to type II error due to sequencing errors and the strictness of this alignment. Results were relatively similar across alignment identities from 0.98 to 1.00; however, it is worth noting that if users want to reduce type II errors resulting in false negative alignments, less stringent alignment identity is favorable (Figure 2(d)).

Having evaluated recall error of the model, we wanted to test the sensitivity of the algorithm to noise, which in clinical practice may be a multitude of novel strains introduced through diet, environment, or other people in contact with patients. To simulate these novel sources, we introduced unrelated donors to the simulated samples. For simplicity, we refer to additional donor reads as a “contaminate source.” Each simulation was contaminated with 20% contaminate sample reads while preserving the ratio of donor and pre-treatment reads in the remainder of the sample (Figure 2(b)). Thus, in the simulations for 50% donor and 50% pre-treatment, we had 20% contaminate, 40% donor, and 40% pre-treatment in the mixtures. Repeating these steps for all donor and pre-treatment pairs, we obtained a measurement of type I error (Figure 2(e)). We found more stringent alignment to be slightly favorable in reducing the false positives. Still, all alignment identities display some increased error, so it is worth noting that engraftment may be overestimated if there are many novel strains in common with the donor.

As seen in Figure 2, there were some outliers in our simulation results that are likely the product of underlying abnormalities in the original dataset. Two samples underestimated engraftment more than the other samples, likely due to their high overlap between the donor and the pre-FMT samples producing more false negatives. The outlying samples, with overestimated engraftment came from participants doing a multi-course of FMT treatment who failed their other treatment. It is possible their pre-FMT samples were more drastically depleted of microbes and therefore produced less robust MAG databases for comparison in our pipeline and therefore more false positives at lower alignment identity.

### MAGEnTa distinguishes successful engraftment in the ulcerative colitis dataset

Applying the pipeline to a real randomized cohort from an ulcerative colitis trial^21^ (clinicaltrials.gov ID NCT03948919) with MTT and a placebo control with 27 participants demonstrated the usefulness of the algorithm. Participants in the study were randomly assigned to receive a daily MTT (*n* = 13) or placebo capsules (*n* = 14) for 8 weeks. Each participant received MTT capsules from a single donor throughout the 8-week dosing period. Material making up donor capsules was sequenced and fed into our pipeline.

Engraftment analysis was completed for the placebo participants even though they did not receive donor material. This was done by assigning each placebo participant a donor (within MAGEnTa) despite not receiving donor material in the trial. The donor material was assigned to each placebo patient in MAGEnTa in the same order MTT participants received their donor assignments (randomly, based on timing of enrollment into the trial). So even though placebo patients did not receive a donor, an estimate of ‘engraftment’ was calculated, allowing for a statistical comparison to donor engraftment with MTT.

**Figure 3.**
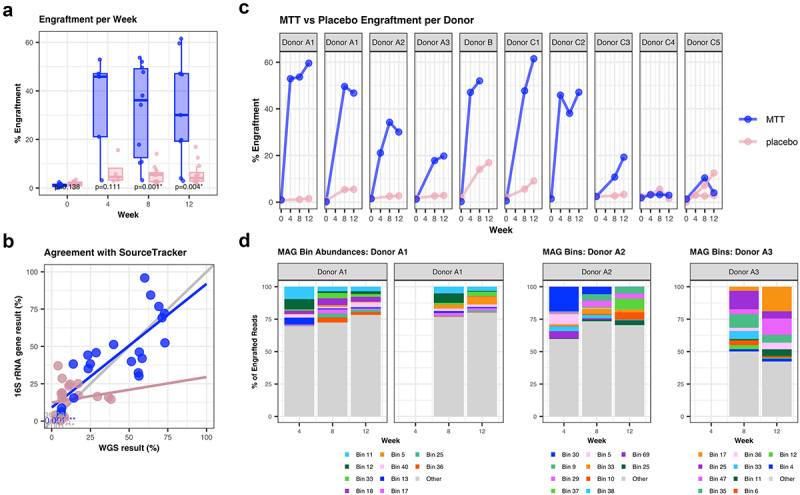
(a) Boxplot showing the percent of donor engraftment per group at each week. The greater spread in the intervention group is likely due to the variances in success of engraftment following the encapsulated MTT course. (b) Agreement between methods per sample. Whole-genome sequencing (WGS) MAGEnTa pipeline results are on the x-axis and 16S rRNA gene SourceTracker results on the y-axis. The intervention samples (blue) have high agreement (Pearson correlation, *p* < 0.001), and placebo samples have no agreement (Pearson correlation, *p* = 0.45); however, poor placebo sample agreement may be poor due to the low engraftment observed. (c) Percentage of donor engraftment for each patient and its matched placebo donor. Donor engraftment in the microbiota transplant therapy (MTT) group is significantly higher at weeks eight and 12 compared to placebo. Three donors were used and participants received donor material from only one donor. MTT is shown in blue and placebo in pink. Some participants are missing samples (predominantly from week 4), but the intervention of daily oral MTT began after collecting the baseline sample at week 0 and ran for the first 8 weeks, collecting samples every 4 weeks. (d) MetaBinner results from binning engrafted metagenome assembled genomes (MAGs) shown as a percentage of engrafted reads for weeks 4, 8, and 12 samples receiving material from Donor A. The top 10 MAG bins are distinctly colored to show relative abundance of reads aligning with these bins across all engrafted reads.

Of the available sequenced samples, two MTT recipients received the capsules of identical donor material (Donor A1, Figure 3(c)). Placebo samples available for sequencing were assigned donor material for comparison based on their order of enrollment, to match timing in the drug intervention group, except one patient, which was randomly assigned a donor lot given the unequal numbering between groups. Aligning each post-MTT sample with the donor and pre-MTT databases, we could discriminate differences between successful and unsuccessful engraftment in the intervention group, with low engraftment estimates per placebo (Figure 3(a–c)). Donor similarity, or engraftment, was significantly higher in patients receiving MTT at weeks eight (*p* = 0.001) and 12 (*p* = 0.004), the primary trial endpoint, compared to placebo (Figure 3(a)). Several time points were missing from week 4, likely affecting power to detect a significant difference between MTT and placebo.

Some intervention participants responded with more engraftment success than others (Figure 3(c), Supp Figure S1A). In those who showed greater engraftment, the proportion or percentage of engrafted strains remained relatively stable through 12 weeks. As expected, in both high engraftment responders and poor engraftment responders, we saw near zero engraftment at baseline, demonstrating the very small overlap between our healthy donors and active ulcerative colitis patients. The control group receiving placebo capsules showed minimal *in silico* engraftment, as expected. Interestingly, two placebo recipients had slightly higher *in silico* engraftment values than others, indicating their random acquisition of strains resembling the healthy donors. As seen before in our sensitivity simulation with the external source, overlap between the donor and novel sources may introduce erroneously inflated engraftment values.

To further validate our pipeline for real-world applications, we compared our results in this cohort to results from 16S rRNA gene data analyzed using SourceTracker software (Figure 3(b)).^9^ Agreement between the 16S rRNA gene SourceTracker results and our WGS MAGEnTa pipeline were in significant agreement (Figure 3(b), linear regression, *p* < 0.001) and visually aligned (Supp. Figure S1B). The agreement between these methods validates our approach, although WGS is well understood to better represent strain-level specificity. We also binned the resulting engrafted MAGs using MetaBinner and visualized the retention of MAG-derived strains in the sample (Figure 3(d)).^22^ MAGEnTa was made to produce engrafted reads and MAGs, enabling users to further explore the engrafted strains by metagenomic approaches such as binning. We then passed these bins into the genome taxonomy database^23–25^ (GTDB) database using the GTDB-Tk tool to associate the engrafted MAG bins with strains in the genome taxonomy database. While taxonomic characteristics for this ulcerative colitis cohort have been previously described,^21^ GTDB-Tk^26^ software found these bins included material from strains of the *Collinsella* genus, specifically the *Collinsella* strain sp002391315 matched bin 11 from Donor A1 (Figure 3(d)). This finding adds further detail to the 16S rRNA results found with SourceTracker which were reported previously.^21^ Further evaluation of the taxonomic and functional characteristics of these engrafted bins and reads are beyond the scope of this methods manuscript but are left up to the researcher. The GTDB-Tk toolkit is only one approach for further evaluation of these engrafted bins and reads, as more detailed functional annotation could be achieved with other metagenomic tools as well.

### Shallow sequencing depth is sufficient, so long as donor and pre-sample are deeply sampled

Since sequencing and computing costs can be affected by the depth of sequencing in WGS data, we evaluated the changes in engraftment value accuracy when subsampling at lower sequencing depths. All samples for this ulcerative colitis cohort were sequenced at a maximum target depth of 24 million reads. Beginning with downsampling the post-MTT samples to depths of 5 million, 2 million, 1 million, and 500,000 reads, we aligned these samples with the full depth databases for each donor and pre-MTT sample. Results of downsampling only the post-MTT samples, shown in Supp. Figure S2(a), show minimal error introduced even at very shallow depths (maximum of approximately 0.2% absolute error). Downsampling the donor and pre-MTT samples prior to building the MAGs databases results in greater error at low depths (Supp Figure S2B), indicating it is best to sequence these samples at high depth, but the post-MTT samples may allow for shallower sequencing to reduce sequencing and computational storage and memory costs.

## Discussion

Simulated and practical applications of MAGEnTa show excellent recall of engrafted strains, maintaining a low false negative rate in accurately estimating donor similarity and quantifying engraftment. Some false positive errors can be introduced by overlap between the donor and the pre-MTT sample or novel strains, and we found that using 100% alignment identity reduced the type I error rate (Figure 2(e)). We further validated the efficacy of our method by comparing donor similarity measures to 16S rRNA gene sequencing results achieved with SourceTracker, attaining high agreement between the methods but with increased strain specificity from the WGS results. Furthermore, we found that our algorithm is robust to shallow depths of WGS, recovering similar values to our full depth analysis in both low-engraftment and high-engraftment samples when the post-MTT samples are sequenced at shallow depths.

As MTT is rapidly being evaluated as a potential treatment for multiple clinical indications, investigators are using varying treatment protocols. Some trials use pre-transplant conditioning regimens, such as antibiotics to increase engraftment. In these cases, there may be more than one patient baseline sample: a sample prior to the pretransplant conditioning method and one after. In these cases, selecting what to use as a ‘baseline’ depends on the goals of the study investigators; the best control would likely be to use both samples. This is because part of the pre-antibiotic microbiome remains intact after antibiotic treatment, and some residual microbiota may rebound even if they are not measurable in the post-antibiotic sample.

The MAGEnTa pipeline assumes only one baseline sample file but allows for customization of these baseline and donor samples, as the user can concatenate multiple sequencing files into a single ‘baseline’ or ‘donor’ sample. In our study, participants of the trial received capsules from the same donor over an 8-week period; thus, multiple stool donations were required to generate capsules for the entire study period. To account for donor variability and to capture all the donor microbiome given to participants, multiple sequencing files from these donor samples were concatenated into a single ‘donor’ file. This approach can be used if multiple sequencing files are available in the event of multiple different baseline samples.

Similarly, if multiple donors are used for a single patient, sequencing files from these donors can also be concatenated into the ‘donor’ file to account for multiple donors. Another variation in MTT protocols is the use of autologous microbiota. To reduce possible false positive errors from a likely significant overlap in the case of an autologous donor, we recommend specifying 100% alignment within MAGEnTa.

In our study, we had trial participants who showed both ‘high’ and ‘low’ engraftment patterns compatible with other studies that find this dichotomy. In our previous study evaluating engraftment with SourceTracker for 16S rRNA sequencing data, we found that engraftment negatively correlated with alpha diversity,^21^ suggesting that having a more ‘available niche’ may increase the chances of higher engraftment. Other factors, such as donor strain richness, baseline recipient factors, and donor-recipient strain complementarity can all impact engraftment success.^9,12,27^

Most placebo patients did not show any ‘donor engraftment’. However, two of the three donors showed low ‘donor engraftment’ in the placebo patients, while one donor (donor B) showed higher placebo ‘engraftment’. In this manner, the appearance of ‘donor engraftment’ in the placebo participants is due to overlap of genomic content in the donor and placebo subject microbiota. Most placebo participants did not show any ‘donor engraftment’ other than one donor (donor B) that had higher ‘engraftment’ than the other donors in placebo participants, indicating likely high similarity of their microbiomes at baseline. This is a limitation of supervised donor tracking when directly using genomic content.

Limitations of the MAGEnTa algorithm depend on the application and expected variation in microbial diversity across samples. In applications with high strain overlap between the participant and donor prior to the study, such as MTT trials where the donors are related to the recipients or when autologous stool is used as the donor, the algorithm may underestimate engraftment due to the disproportionate number of ambiguous strains in the sample. The algorithm is also dependent on reliably deep WGS for the original donor and pre-MTT patient samples to enable assembly of representative MAGs. Therefore, if funds are limited, it is better to more deeply sequence the baseline and donor samples than the post-transplant samples. MAGEnTa also only estimates the source environment proportions and MAG-level sequence assignments but does perform the full taxonomic and functional annotation of the source MAGs; this additional information can be added using a variety of existing methods for sequence, gene, and MAG annotation.

MAGEnTa is intended as a preliminary step in evaluating engraftment following MTT, applicable in any MTT study using WGS. The pipeline produces reliable values for community-level source tracking and engraftment of strains and may be applied to timeseries data to study the kinetics of engraftment in a subject. For instance, persistence of engrafted MAGs from the donor can be measured during an MTT course, as shown in Figure 3(d). Furthermore, reads, MAGS, or bins determined as engrafted can be analyzed with other metagenomic techniques to characterize the taxonomy of engrafted strains, to evaluate beta diversity at the strain level, and to identify functional profiles of engrafted strains associated with clinical outcomes. Future improvements to the pipeline could leverage more capabilities of metagenomic analysis via integration with other tools, incorporating more taxonomic and functional annotations to more deeply characterize the engrafted and un-engrafted strains.

We hope to see the MAGEnTa pipeline applied in future studies evaluating MTT in a variety of indications, to provide reliable quantitative evaluation of engraftment and fine-grained strain-level analysis.

## Materials and methods

### Simulated engraftment dataset

To generate a simulated dataset representing levels of engraftment, we used publicly deposited WGS data from a previous study of FMT treatment for *C. difficile* infections.^13^ From this cohort, we obtained 16 donor and patient pairs for which engraftment was listed as successful. We then simulated post-treatment samples by subsampling the donor and pre-treatment FASTQ files, achieving an even depth of 8.75 million reads per sample after quality trimming. In increments of 10%, we obtained 11 simulated post-treatment samples per pair constituting every level of engraftment from 0% donor to 100% donor. For example, with the 20% donor sample, we obtained 0.8 × 8.75 M = 7 M reads from the pre-treatment sample and 0.2 × 8.75 M = 1.75 M reads from the corresponding donor sample. We elected to use real reads as the basis for these simulations to incorporate a reasonable amount of noise expected in microbiome samples obtained during an FMT trial. These simulated samples are known to have exactly 0%, 10%, … 100% donor reads contained in them, providing a simple true positive measure for accuracy and sensitivity evaluations.

### Ulcerative colitis trial study samples

We used WGS samples from a randomized, double-blinded, placebo-controlled MTT trial in patients with mild-to-moderate ulcerative colitis.^21^ The primary endpoint of this trial was engraftment of donor microbiota at week 12 (clinicaltrials.gov ID NCT03948919; IND 18,682). The study was described in detail previously.^9^ Briefly, the MTT regimen consisted of 8 weeks of daily administration of two capsules of compound MTP-101C,^28^ each capsule containing 1–2 × 10^11^ bacteria with ≥40% viability determined by a membrane integrity assay. The placebo capsules contained trehalose and carboxymethylcellulose and were indistinguishable from MTT capsules. Both active drug and placebo were manufactured by the University of Minnesota Microbiota Therapeutics Program using Good Manufacturing Practice protocols. A baseline stool sample, followed by weekly stool samples were collected for each patient from weeks 1 to 8 (active drug dosing weeks) and at week 12 (4 weeks after the last dose). Donor material that made up capsules given to MTT participants was sequenced, and donors were not genetically related to participants. Thirteen participants were randomized to MTT, and 14 were randomized to placebo, and one participant from the placebo group and one patient from the MTT group did not provide sufficient follow-up for engraftment analysis. Each patient randomized to the MTT group received material from the same donor for the entire duration of the 8-week dosing period. Some samples were not available for WGS due to DNA concentrations that were too low. These include: 1) one and two missing baseline samples, 2) seven and nine missing week 4 samples, 3) two and one missing week 8 samples, and 4) three and no missing week 12 samples in the MTT and placebo group (respectively for all). Week 4 had predominantly more missing samples, as stool microbiota this week were collected from swabbing toilet paper, rather than swabbing a whole stool sample. The missing baseline, pre-treatment samples completely precluded engraftment analysis for those ulcerative colitis participants.

### Engraftment design

To make enough donor material to last each participant the entire 8 weeks of treatment, multiple stool donations from the same donor were utilized. For each stool preparation, DNA was sequenced, therefore, when setting up MAGEnTA, there were several sequenced files from the same donor for each participant. To fully capture the donor material each participant received, sequenced files from different preparations of donor material used during MTT were concatenated into a single ‘donor’ file.

To control for ‘donor engraftment’ in placebo participants, we assigned them a ‘donor’ file within MAGEnTa that was the same donor sequence file assigned to a participant that received MTT within MAGEnTa. The first participant enrolled into the placebo group was matched to the same donor file within MAGEnTa as the first patient enrolled into the MTT group, and so on, to allow for equal representation of donors between both groups.

### DNA extraction

Collected samples were stored in an ethanol solution at −20 degree Celsius until DNA extraction and were then stored at −80 degree Celsius until metagenomic sequencing. DNA was extracted using the DNeasy PowerSoil Kit (QIAGEN, Hilden, Germany) per the manufacturer instructions from stool swabs (Sarstedt tube and swab, Newton, NC, USA) in 3 mL of 95% ethanol, and sequencing was completed by the University of Minnesota Genomics Center.

### Library creation

To assess DNA sample quality and protein contamination, samples were quantified using a fluorimetric PicoGreen assay and a Nanodrop (greater than 1 ng/µL to pass quality control) Genomic DNA samples were converted to Illumina sequencing libraries using the Seqwell purePlex DNA library preparation kit (Cat. # 301067–301070), and the purePlex workflow was completed in accordance with the protocol. Five to 50 ng of input DNA underwent two tagging reactions, the first with a unique i7 and the second containing the i5 and a normalization reagent. After stopping the reactions, the libraries were pooled in batches of 24, purified, and then amplified with PCR. Final pools were validated for size distribution using capillary electrophoresis and quantified using PicoGreen fluorimetry.

### Cluster generation and sequencing

Pooled libraries were denatured and diluted to the appropriate clustering concentration and were loaded onto the NovaSeq paired-end flow cell. Clustering was carried out onboard the instrument. Once clustering was complete, sequencing was completed using Illumina’s two-color SBS chemistry, and upon read completion, one, two separate, eight or 10 base pair index reads were performed. Clustered library fragments were re-synthesized in reverse direction to produce the template for paired end read two. For each sequenced sample, ≥2,250 million cluster count pass filter reads for each lane were generated. Samples were sequenced to an average of 24 million reads per sample using the NovaSeq 6000 (Illumina) sequencer.

### Primary analysis and de-multiplexing

Base call (.bcl) files for each sequencing cycle were generated by Illumina Real-Time Analysis software, and the base call files and run folders were maintained on servers at the Minnesota Supercomputing Institute. The primary analysis and de-multiplexing were performed using the Illumina bcl-convert v4.0.3. The result of the bcl-convert workflow is a de-multiplexed, paired FASTQ files generated containing 150 bp length reads per sample.

### Processing metagenomic data

Human sequence read removal, quality filtering, and adapter trimming were completed with the KneadData command integrated into the bioBakery software package.^17^ Default values of sliding window 4 to 20 and a minimum length of 75 base pairs were used for the Trimmomatic step (Bolger et al.).^29^ The paired, filtered FASTQ files produced by KneadData were passed to the start of our pipeline, which assumes quality control is complete. Our pipeline produces a list of engrafted reads and engrafted MAGs. To demonstrate how these results may be used in downstream analyses we binned MAGs into representative genomes using the ensemble binning software MetaBinner with minimum contig length 1000 and kmer length 4 to construct the composition profile.^22^

### Statistical methods

To evaluate the type I and type II error differences between alignment identities in our simulations, we used a Student’s t-test for paired group-wise comparisons of the three alignment identities (0.98, 0.99, 1.00). Given the non-parametric distribution of the intervention group, we evaluated randomization group differences per week with a Mann–Whitney U-test. To compare our engraftment results to those obtained from SourceTracker, we used a Pearson correlation test to evaluate if the correlation coefficient is statistically significant. When comparing to SourceTracker, we evaluated the Pearson coefficient within the intervention and placebo groups both independently and together.

## Supplementary Material

Supplemental Material

## Data Availability

Our pipeline, MAG Engraftment Tracker (MAGEnTa), is available at https://github.com/knights-lab/MAGEnTa.
