# Supplementary material for: Metagenomic source tracking after microbiota transplant therapy
Source: Gut Microbes. 2025 Apr 14;17(1):2487840. doi: 10.1080/19490976.2025.2487840 (PMC12005403; doi:10.1080/19490976.2025.2487840)
Supplement: Supplemental Material [file KGMI_A_2487840_SM3004.docx]

**Figure S1.** (A) Engraftment lines per sample, colored by randomization group (MTT in blue, placebo in pink). Mean engraftment lines per group are shown with dashed, bolded lines. (B) Comparing results from the SourceTracker tool and our pipeline results per sample, organized by donor preparation and randomization group.


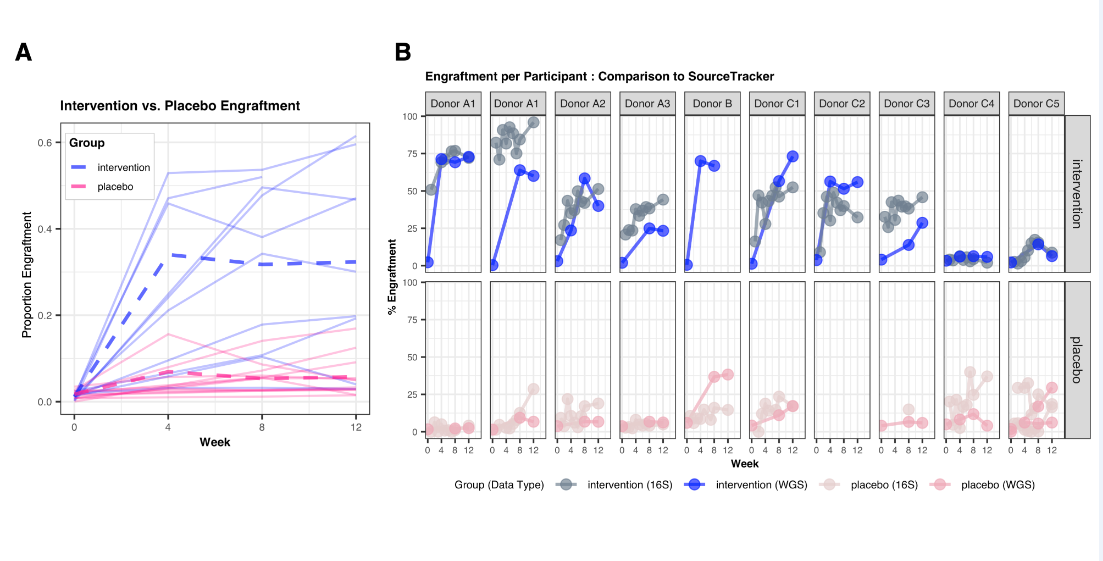


**Figure S2.** (A) Varied sequencing depths for post-treatment samples in our ulcerative colitis dataset, using full depth databases for the donor and pre-treatment samples. Using the full depth (20M reads) results as truth, distances of each sample at each depth to the truth are shown. Lines connect each sample to itself at each respective depth. (B) A case study of a single post-treatment set from week 4 (circle), week 8 (square) and week 12 (triangle) is tested at varied sequencing depth for all samples, including the donor and pre-treatment samples. Greater error is introduced than using full depth donor and pre-treatment samples, indicating the importance of deeply sequencing the donor and pre-treatment samples forming MAGs databases in the algorithm. Note that down sampling was completed after trimming and quality read removal.

**
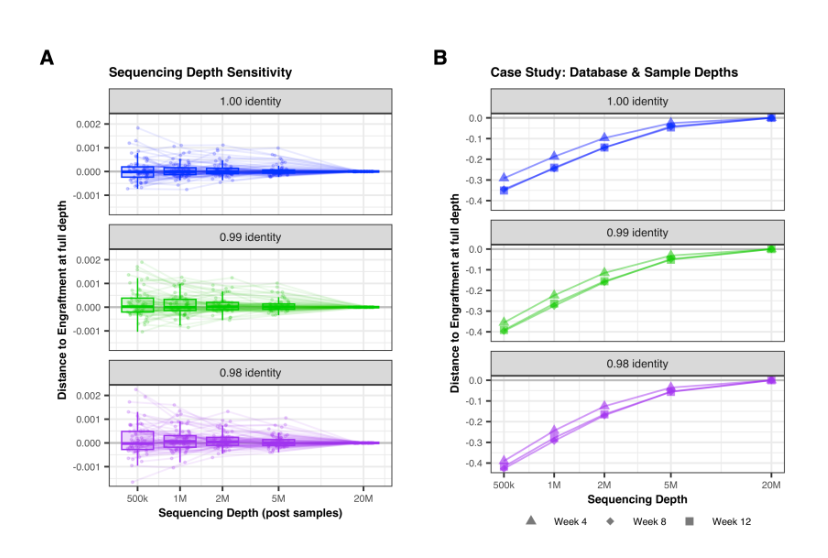
**
